# Long-term tumor control following gamma-knife radiosurgery of recurrent or residual pituitary adenomas: a population-based cohort study

**DOI:** 10.1007/s00701-024-06380-9

**Published:** 2024-11-30

**Authors:** Alexander Gabri, Felicia Lindberg, Helena Kristiansson, Michael Gubanski, Charlotte Höybye, Martin Olsson, Petter Förander, Simon Skyrman, Bodo Lippitz, Alexander Fletcher-Sandersjöö, Jiri Bartek

**Affiliations:** 1 Department of Neurosurgery, Karolinska University Hospital, Eugeniavägen 3, 171 76 Stockholm, Sweden; 2 Department of Clinical Neuroscience, Karolinska Institute, Stockholm, Sweden; 3 Department of Endocrinology, Karolinska University Hospital, Stockholm, Sweden; 4 Department of Molecular Medicine and Surgery, Karolinska Institute, Stockholm, Sweden; 5 Department of Neurosurgery, Rigshsopitalet, Copenhagen, Denmark

**Keywords:** Pituitary adenoma, Gamma knife radiosurgery, Stereotactic radiosurgery, Neurosurgery

## Abstract

**Background:**

Pituitary adenomas (PAs) often require adjuvant Gamma Knife radiosurgery (GKRS) due to partial resection or postoperative recurrence. The purpose of this study was to evaluate the long-term efficacy of GKRS for such cases.

**Methods:**

Population-based, observational cohort study of patients who underwent GKRS for postoperative residual or recurrent PAs between 1999 and 2020. We assessed long-term progression-free survival (PFS), identified predictors of tumor growth, and documented adverse radiation events (AREs).

**Results:**

147 patients were included, with a median follow-up time of 8.1 years. Following GKRS, the 5-year and 10-year PFS was 86% and 68%, respectively, with a median PFS of 18.7 years. Somatotrophic adenomas showed a significantly lower risk of tumor progression in the regression analysis (OR 0.11, *p* = 0.003). Hypopituitarism was the most common ARE, affecting 32% of patients.

**Conclusion:**

GKRS is an effective treatment option for recurrent or residual PAs, offering substantial long-term control. However, the risk for AREs, particularly hypopituitarism, is not negligeable.

## Introduction

 Pituitary adenomas (PAs) are benign tumors that originate from the anterior lobe of the pituitary gland. Although they account for up to 15% of all intracranial masses [14], clinically significant PAs only occur in approximately 1 per 100,000 people [3]. The primary treatment for symptomatic PAs is typically surgical removal, most often using a transsphenoidal technique. However, 26% of cases result in partial resection only [2]. Additionally, even after gross total resection (GTR), tumor recurrence occurs in approximately 25% of cases within 10 years [8]. For patients with residual or recurrent PAs, Gamma knife radiosurgery (GKRS), is often used as a possible adjunctive treatment method in these cases [5, 10, 11].

Several studies have explored the effectiveness of GKRS in residual or recurrent PAs, reporting progression-free survival (PFS) rates from 53 to 100% across follow-up periods from 3.4 to 15 years (median follow-up 5.6 years) (Table 1). However, the clinical relevance of these findings is limited by the relatively short follow-up times, leaving a gap in our understanding the long-term efficacy of adjuvant GKRS for PAs.

**Table 1:** Previous studies on tumor control after GKRS of PAs

Reference	Patients (*n*)	Tumor type	Years follow-up (median)	Estimated PFS	Treatment indication
Deng [4]	148	NFPA	5.4	5 years: 88% 10 years: 74%	Residual or Recurrent
Dumot [5]	869	NFPA	3.7	5 years 95.5% 10 years 88.8%	Residual or Recurrent
Gopalan [6]	48	NFPA	6.7	5 years: 87% 10 years: 76% 15 years: 76%	Residual or Recurrent
Jezkova [12]	28	LH-secreting	11.7	5 years: 100% 10 years: 100%	Residual
Kotecha [13]	2671	NFPA	4	5 years 94% 10 years 83%	Residual or Recurrent
Lee [16]	41	NFPA	4	5 years: 94% 10 years: 85%	Residual or Recurrent
Losa [17]	54	NFPA	3.4 (mean)	5 years: 88%	Recurrent
Mathieu [18]	2289	F-PA	NA	Acromegaly 97% Cushing’s 92% Prolactinoma 93%	Residual or Recurrent
Narayan [20]	111	Mixed (22% F-PA)	4	5 years: 93% 10 years: 64%	Residual or Recurrent
Park [21]	125	NFPA	5.2	5 years: 94% 10 years: 76%	Residual or Recurrent
Rim [22]	60	Mixed (58% F-PA)	5.7	10 years F-PA 66% 10 years NFPA 96%	Residual
Shaaban [23]	360	NFPA	7.95	5 years 93% 10 years 86% 15 years 69%	Residual or Recurrent
Starke [24]	140	NFPA	4.2	5 years: 97% 10 years: 87%	Residual or Recurrent
Sun [25]	204	NFPA	7.2	5 years: 95% 10 years: 92% 15 years: 81%	Residual or Recurrent
Yu [27]	81	NFPA	5.6	5 years: 95% 10 years: 84%	Primary treatment

*GKRS* Gamma knife radiosurgery, *PA *pituitary adenoma, *PFS* progression-free survival, *F-PA* functioning pituitary adenoma, *NFPA* non-functioning pituitary adenoma

In addition to the above, predictors of continued tumor growth in these cases remains unclear. Although genetic markers might account for progression in a subset of patients [26], the effect of demographic, radiographic and treatment-related factors remain uncertain. The literature suggests that tumor size exceeding 5mL [6, 20, 25] and a marginal prescription dose (MPD) below 12 Gray (Gy) may be associated with increased risks for relapse or progression following GKRS [4, 6, 20]. However, the available data is sparse, perpetuating a knowledge gap in this area.

In light of the above, the primary aim of this study was to assess long-term PFS following GKRS of postoperative recurrent or residual PAs. Secondary aims included identifying predictors of post-GKRS tumor growth and the incidence of adverse radiation events (AREs).

## Methods

### Study design and patient selection

This was a retrospective population-based cohort study performed at the Department of Neurosurgery, Karolinska University Hospital, Stockholm, Sweden. We included adult patients with PAs who underwent postoperative GKRS between 1999-05-01 and 2020-06-30. Patients with insufficient or missing follow-up data were excluded, as well as patients residing outside the Stockholm region due to challenges in accessing comprehensive follow-up data. These patients often receive postoperative care and long-term monitoring at their local hospitals, making it difficult to obtain consistent and complete medical records necessary for this study. Ethical considerations, including patient consent and compliance with data protection regulations, further limited our ability to include these patients. The patients were identified from the Leksell Gammaplan^®^ (Elekta Instruments Inc.) database, where all patients treated with GKRS are registered and treatment information is saved. Medical records, GKRS data, and imaging data were reviewed using the TakeCare ^®^ health record software (CompuGroup Medical Sweden AB, Farsta, Sweden) and the Leksell Gammaplan^®^ 10.1.1 (Elekta Instruments Inc.). Data collected from patients’ medical records included: date of birth, sex, date of index tumor surgery, tumor type, radicality of index surgery, date of first GKRS, indication for GKRS, tumor volume at first GKRS, MPD, date of last follow-up MRI, date of post-GKRS tumor growth (radiological growth assessed by neuroradiologist), AREs (baseline status equals the day of first GKRS), date and cause of death. The study was approved by the Swedish Ethical Review Authority (Dnr: 2017/1760-31/1), who waived the need for informed consent.

### GKRS treatment and follow-up

The indication for GKRS in this study cohort was postoperative residual PAs, or PAs that had recurred following initial GTR. In all cases, patient eligibility for GKRS was determined by a multidisciplinary board. GKRS was typically not offered if renewed surgery was required due to significant mass effect, if the estimated radiation dose to critical brain structures was deemed too high, or if watchful waiting was a viable management option.

For prolactinomas, dopamine agonist therapy was typically discontinued 2 weeks prior to GKRS. After the procedure, blood prolactin levels were monitored, and the decision to restart medication was made based on the patient’s hormonal response, with the aim of avoiding the need for further medical treatment if prolactin levels remained stable. In the case of acromegaly, somatostatin analogs were stopped 4 weeks prior GKRS. Post-treatment, growth hormone and insulin-like growth factor 1 levels were tracked, and somatostatin therapy was resumed only if necessary, depending on the patient’s endocrinological status.

As to the GKRS procedure, a stereotactic frame was affixed to the patients’ skull under local anaesthesia. After frame fixation, a stereotactic MRI was performed for delineation of target and adjacent structures. Based on the MRI, a treatment plan was made and GKRS was performed as a single session with the aim of attaining highest possible precision with a MPD of 15 Gy or higher without exceeding 8 Gy toward sensitive structures (dose coverage of at least 99% was accepted). GKRS procedures were performed using Leksell Gamma Knife^®^ C (1999–2010), Perfextion^®^ (2010–2018), and Icon^®^ (2018–2021). Patients were monitored for two hours in the neurosurgical ward before being discharged. Follow-up was managed by endocrinologists who continuously follow the patients and refer these to ophthalmologists and neurologists when needed. Radiological follow-up included MRIs at 1, 2, 5, 8 and 10 years after GKRS, with individual variations and extended follow-up determined by the multidisciplinary tumor board on a case-by-case basis.

### Statistical analysis

The normality of continuous data was evaluated using the Shapiro-Wilks test, which revealed significant deviations from a normal distribution pattern for all continuous variables (Shapiro-Wilks test p-value < 0.05). Therefore, continuous data were presented as medians with interquartile ranges, and categorical data were reported as counts with proportions. A Kaplan-Meier plot was used to illustrate PFS, accompanied by the number of patients at risk. Logistic regression analysis was used to determine predictors of tumor growth after GKRS, with explanatory variables including age, treatment indication (residual tumors as reference), sex, tumor type (non-functioning pituitary adenoma (NFPA) as reference), tumor volume, MPD, and time between surgery and GKRS. All analyses were performed using the statistical software R (R version 4.1.2). Statistical significance was set at *p* < 0.05

## Results

### Baseline and treatment data

**Fig. 1:**
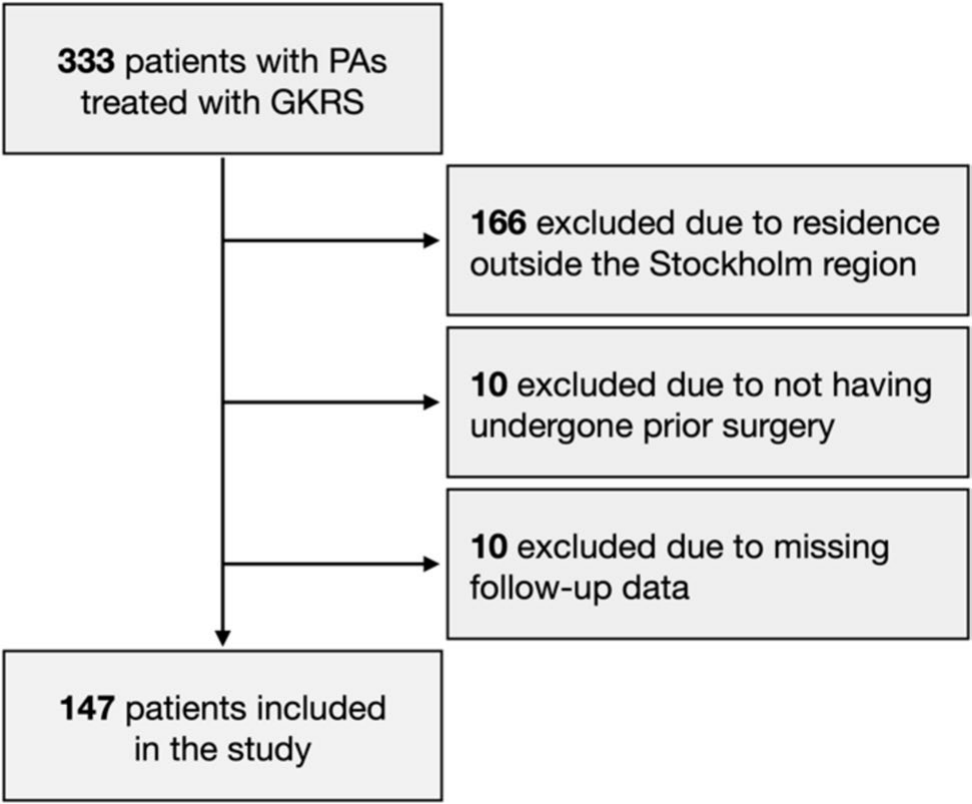
Patient inclusion process. Abbreviations: GKRS = Gamma knife radiosurgery

Out of the 333 patients who underwent GKRS for their PAs during the study period, 186 were excluded due to residence outside of the Stockholm Region, not having received initial surgical resection, or inadequate follow-up data (Fig. 1). Thus, 147 patients were included in the study. The cohort had a median age of 52 years (40–63 years), a balanced sex distribution, and 65 (44%) had functioning pituitary adenomas (F-PAs).

Of the included patients, 82 (56%) underwent GKRS for stationary residual tumors, 48 (33%) for residuals that had progressed on serial imaging, and 17 (12%) due to tumor recurrence following initial GTR. The median MPD was 22 Gy (20–25 Gy), and the median time between index surgery and GKRS was 1.7 years (0.8–4.7 years). The median radiological follow-up time was 8.1 years (4.5–12 years) (Table 2).

**Table 2:** Baseline, treatment, and outcome data

Variable	All patients (*n* = 147)
Age (years)	52 (40–63)
Male sex	80 (54%)
Tumor volume (cm^3^)	0.7 (0.4–1.5)
Hormonal activity	
NFPA	82 (56%)
GH secreting tumor	39 (27%)
ACTH secreting tumor	17 (12%)
PRL secreting tumor	8 (5.4%)
TSH secreting tumor	1 (0.7%)
GKRS indication	
Stationary residual tumor	82 (56%)
Progressing residual tumor	48 (33%)
Tumor recurrence following gross total resection	17 (12%)
Years from surgery to GKRS	1.7 (0.8–4.7)
Marginal prescription dose (Gray)	22 (20–25)
Follow-up time (years)	8.1 (4.5–12)
Outcome	
Tumor growth	38 (26%)
Time to progression (years)	5.3 (2.3–8.0)
5-year PFS	86%
10-year PFS	68%
Median PFS (years)	18.7
Death	22 (15%)
Death due to tumor	3 (2.0%)
Adverse radiation event	
Hypopituitarism	47 (32%)
Vision perimetry deficits	9 (6.1%)
Cranial nerve palsy	7 (4.8%)
Headache	4 (2.7%)

Data presented as median (interquartile range) or number (proportion). *ACTH *adrenocorticotropic hormone, *GH *growth hormone,* GKRS* Gamma knife radiosurgery, *NFPA* non-functioning pituitary adenoma, *PFS* progression-free survival, *PRL* prolactin, *TSH *thyroid-stimulating hormone

### Outcomes

**Fig. 2:**
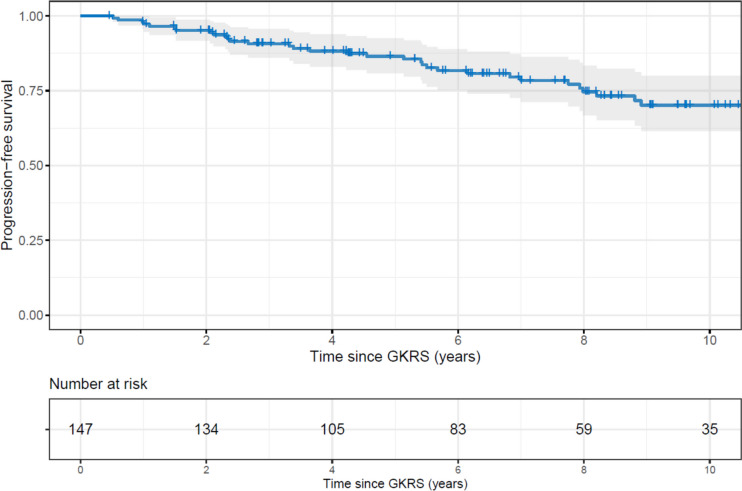
Kaplan Meier plot of tumor growth following gamma knife treatment pooled, including number at risk over time and progression-free survival time with confidence interval

In total, 38 patients (26%) showed tumor growth after GKRS, with a median time to progression of 5.3 years (2.3–8.0 years) (Table 2). The 5-year PFS rate was 86% (95% CI: 80 – 92%), while the 10-year PFS was at 68% (95% CI: 58 – 78%) (Fig. 2). The median PFS was 18.7 years, although at this point only 6 patients remained under observation. There were no significant differences in PFS based on the indication for treatment (*p* = 0.840), or F-PA vs. NFPAs (*p* = 0.190).

In the logistic regression analysis predicting tumor growth, somatotrophinomas were associated with a significantly reduced risk of growth (OR = 0.13, *p* = 0.008). No additional predictors of tumor growth were identified (Table 3).

**Table 3:** Univariable logistic regression predicting post-GKRS tumor growth

Variable	OR (95% CI)	*p* value
Age (years)	1.00 (0.98–1.02)	0.921
Male sex	1.62 (0.77–3.53)	0.211
Treatment indication		
Stationary residual tumor	ref	ref
Progressing residual tumor	0.76 (0.3–1.72)	0.521
Tumor recurrence following GTR	0.79 (0.2–2.50)	0.704
Endocrine activity		
Non-functioning pituitary adenoma	ref	ref
ACTH secreting tumor	1.69 (0.56–4.94)	0.339
GH secreting tumor	0.13 (0.02–0.48)	**0.008**
PRL secreting tumor	4.03 (0.92–20.9)	0.070
Tumor volume (cm^3^)	1.15 (0.89–1.48)	0.262
Marginal prescription dose (Gy)	0.94 (0.87–1.01)	0.150
Years between surgery and GKRS	0.96 (0.86–1.005	0.395

*ACTH *adrenocorticotropic hormone, *CI* confidence interval, *GH* growth hormone, *GKRS* Gamma knife radiosurgery, *GTR * gross total resection, *OR *odds ratio, *PRL *prolactin, *TSH *thyroid-stimulating. Bold text in the *p*-value column indicates a statistically significant correlation (*p* < 0.05)

Hypopituitarism (full or partial) was the most prevalent ARE, affecting 47 patients (32%), followed by vision perimetry deficits in 9 patients (6.1%), other cranial nerve palsies in 7 patients (4.8%), and treatment-related headaches in 4 patients (2.7%). Throughout the follow-up 29 patients passed away. Causes of death were malignant PA (*n* = 3), other malignancies (*n* = 6), respiratory or circulatory failure (*n* = 18), and trauma (*n* = 2) (Table 2).

## Discussion

In this retrospective population-based study, GKRS for postoperative residual or recurrent PAs was associated with a 5-year PFS of 86% and a 10-year PFS of 68%, with a median PFS of 18.7 years. Somatotrophic adenomas were associated with a significant lower rate of tumor growth, while GKRS indication (stationary residual vs. progressing residual vs. recurrent tumors) did not affect tumor control. The most common ARE was hypopituitarism, affecting 47 (32%) patients. The study’s findings regarding PFS align with current literature, which reports PFS from 54–100% at the 5-year mark and 53–100% at the 10-year mark [4, 18, 20, 25]. While this study’s PFS estimates fall within the mid-range of these published intervals, there are variations in other treatment-related factors compared to the proposed averages. The time after GKRS to tumor progression or recurrence among the 38 patients (26%) reached the upper limit suggested in the literature, with a median of 5.3 years [5, 13, 18, 23]. Furthermore, the median duration between primary surgery and GKRS in our cohort was 1.7 years, contrasting with the under-one-year time frame reported in referenced literature. These disparities largely stem from the limited exclusion criteria applied in this study, where there was no lower limit on follow-up time among patients, and all patients who were selected for and treated with GKRS were included. Consequently, our study cohort represents a population-based sample. Supported by a consistent treatment approach throughout the study period, this study provides support for its PFS estimates, with a median PFS of 18.7 years. However, it should be noted that there is an inherent uncertainty in the estimates due to the retrospective nature of the study and the limited number of patients followed for more than 10 years The existing literature on PFS for F-PAs is less extensive compared to NFPAs, as most studies tend to focus on endocrinological endpoints rather than radiological ones, as evidenced by Albano et al.’s meta-analysis [1]. Whether F-PAs exhibit differential sensitivity to GKRS compared to NFPAs remains a subject of debate. Some studies have reported higher PFS rates favouring F-PAs, while others have suggested otherwise [17, 20]. This complexity is further compounded by variations in treatment protocols with higher radiation doses typically applied to secreting tumors to achieve endocrinological control [7]. Among the potential predictive factors for tumor progression or recurrence that were studied, somatotrophic adenomas had a lower risk for post-GKRS growth, while these also generally received higher MPD than other tumors in our cohort. This may in return reflect the outcome of better radiological tumor control among somatotrophic adenomas in our dataset. The most prevalent ARE following GKRS was full or partial hypopituitarism, followed by vision perimetry deficits, other cranial nerve paresis, and headache. Hypopituitarism is a well-known complication following interventions involving the pituitary gland, with a cumulative risk increasing over time and an average incidence of up to 60% during the first 10 years after GKRS [4, 9, 18, 27]. However, establishing whether hypopituitarism results from GKRS or would have occurred regardless due to previous interventions can be challenging since patients are rarely treated with only a single modality and/or in a single session. Headache, on the other hand, is a nonspecific complication with a proposed cause being stretching of the dural sheet [14]. Cranial nerve paresis and vision perimetry deficits have been strongly linked to a radiation threshold that nerves can withstand [15, 19], resulting in a decreased incidence in modern era series as compared to other complications where preventive measures are less feasible. Nevertheless, despite the known correlation between radiation dose and nerve damage, there are cases where the tumour’s location is challenging albeit GKRS still being indicated. Coupled with individual variations in sensitivity to radiation, this has led to reported prevalence rates of vision perimetry deficits and cranial nerve paresis after GKRS ranging from 0–8% and 3–14% respectively [4, 13, 20, 27]

### Strengths and limitations

This study’s strengths lie in its population-based design within a single healthcare system, ensuring uniformity in patient management and follow-up procedures. As the treatment strategy for PAs was the same throughout the study, there was no need to adjust for confounders attributed to screening, diagnostics, or treatment plan. However, excluding patients residing outside Stockholm may introduce selection bias and limit the generalizability of our findings. Future studies involving multicentre collaborations are necessary to confirm the applicability of our results to a broader population. Furthermore, as this study focused on PFS and AREs the radiological data presented mainly assists these investigations. The exclusion of usual metrics for evaluation of radiotherapy quality (e.g. conformity indices) without a substitute due to its lower sensitivity in evaluating PA treatment plans may weaken the method and should be considered in future studies examining GKRS toward the pituitary gland. The study also did not establish a definitive measure for PA progression, relying on neuroradiologist assessments instead of reproducible radiological or endocrinological criteria in preference of reflecting the clinical setting. Although preserving internal validity it affects the study’s external validity which illuminates the absence of an international standard for PA-progression post-GKRS. Lastly, the follow-up consisted of a multidisciplinary set of specialists of at least neurosurgeons and endocrinologists, with additional specialists consulted when needed for optimal therapy. This extensive, usually life-long clinical follow-up decreases the necessity of radiological controls with time as the tumor is deemed stable. Therefore, radiological control periods longer than 10 years are rare, introducing uncertainty for long-term PFS estimates.

## Conclusion

Following initial surgical resection, GKRS of recurrent or residual PAs was associated with a 5-year PFS of 86% and a 10-year PFS of 68%. Treatment-induced hypopituitarism, affecting 32% of patients, was the most common ARE.

## Data Availability

No datasets were generated or analysed during the current study.

## Abbreviations

ACTH: adrenocorticotropic hormone
ARE: adverse radiation event
F-PA: functioning pituitary adenoma
GH: growth hormone
GKRS: Gamma Knife radiosurgery
GTR: gross total resection
GY: Gray (SI)
MRI: magnetic resonance imaging
MPD: marginal prescription dose
NFPA: non-functioning pituitary adenoma
PA: pituitary adenoma
PFS: progression-free survival
PRL: prolactin
TSH: thyroid-stimulating hormone
